# Therapeutic efficacy of ethanolic extract of *Aerva javanica* aerial parts in the amelioration of CCl_4_-induced hepatotoxicity and oxidative damage in rats

**DOI:** 10.3402/fnr.v60.30864

**Published:** 2016-04-07

**Authors:** Ahmed H. Arbab, Mohammad K. Parvez, Mohammed S. Al-Dosari, Adnan J. Al-Rehaily, Khalid E. Ibrahim, Perwez Alam, Mansour S. Alsaid, Syed Rafatullah

**Affiliations:** 1 Department of Pharmacognosy, College of Pharmacy, King Saud University, Riyadh, Saudi Arabia; 2 Department of Pathology, King Khalid University Hospital, King Saud University, Riyadh, Saudi Arabia; 3 Medicinal, Aromatic and Poisonous Plants Research Center, College of Pharmacy, King Saud University, Riyadh, Saudi Arabia

**Keywords:** *Aerva javanica*, hepatoprotection, oxidative stress, DCFH, CCl_4_, rutin, liver diseases

## Abstract

**Background:**

Liver diseases, the fifth most common cause of global death, can be metabolic, toxin-induced, or infective. Though approximately 35 Saudi medicinal plants are traditionally used to treat liver disorders, the hepatoprotective potential of *Aerva javanica* has not been explored.

**Objective:**

To investigate the antioxidative and hepatoprotective effect of *Aerva javanica*.

**Design:**

Total ethanol extract of *A. javanica* aerial parts was prepared and tested on DCFH-toxicated HepG2 cells *ex vivo*, and in CCl_4_-injured Wistar rats *in vivo*. MTT assay was used to determine cell viability and the serum biochemical markers of liver injury as well as histopathology was performed. *In vitro* 1,1-diphenyl-2-picrylhydrazyl and β-carotene free-radical scavenging assay and phytochemical screening of the extract were done. Furthermore, *A. javanica* total extract was standardized and validated by high-performance thin layer chromatographic method.

**Results:**

MTT assay showed that, while DCFH-injured cells were recovered to ~56.7% by 100 µg/ml of the extract, a 200 µg/ml dose resulted in hepatocytes recovery by ~90.2%. Oral administration of the extract (100 and 200 mg/kg.bw/day) significantly normalized the serum glutamate oxaloacetate transaminase, serum glutamate pyruvate transaminase, gamma-glutamyl transferase, alkaline phosphatase, bilirubin, cholesterol, high-density lipoprotein, low-density lipoprotein, very-low-density lipoprotein, triglyceride, and malondialdehyde levels, including tissue nonprotein sulfhydryl and total protein in CCl_4_-injured rats. In addition, the histopathology of dissected liver also revealed that *A. javanica* cured the tissue lesion compared to silymarin treatment. *In vitro* assays revealed strong free-radical scavenging ability of the extract and presence of alkaloids, flavonoids, tannins, sterols, and saponins where rutin, a well-known antioxidant flavonoid, was identified.

**Conclusions:**

Our findings demonstrate the potential of *A. javanica* in the attenuation of *ex vivo* and *in vivo* hepatotoxicity and oxidative damage. This further suggests its therapeutic value in various liver diseases. However, isolations of the active principles, their mechanisms of action, and other therapeutic contributions remain to be addressed.

Liver is one of the most vital organs of the body, considered to be the metabolic ‘engine room’. Major functions of the liver include carbohydrate, protein, and fat metabolism as well as secretion of bile, and the storage and detoxification of several drugs and xenobiotics. Liver diseases can be classified as acute or chronic hepatitis (inflammatory liver diseases), hepatosis (non-inflammatory liver diseases), and cirrhosis. Liver disorders can be attributed to toxins like aflatoxin, chlorinated hydrocarbons, and certain drugs. Other factors include infectious pathogens such as hepatitis viruses (1) and alcohol consumption (2). Drug-induced liver toxicity accounts for approximately one-half of the cases of acute liver failure (3). Currently, there is a real need for effective therapeutic agents, especially natural products with a minimal incidence of toxic effects. Despite the significant popularity of several mono- and poly-herbal preparations for liver diseases (4), only few (e.g., silymarin) have been approved for protection or treatment of liver disorders (5). Nevertheless, in recent years, many phytoproducts have been investigated against hepatotoxin-induced liver damage (6–10).

The genus *Aerva* belongs to the plant family *Amaranthaceae*, and there are approximately 28 species in this genus (11). *Aerva javanica* is an erect, much-branched perennial herb that is a native of Africa and also distributed in various parts of the world. Different parts of the plant have wide applications in folk medicine, for example the whole plant is used for chest pain, diarrhea, and as a diuretic and demulcent. A decoction prepared from the aerial parts of *A. javanica* is used as a gargle to cure gum swelling and toothache. Though the seeds are used for headache relief, paste from the flowers and leaves is used externally to heal the wounds and the inflammation of joints (12). Also, it is used for dysentery, gonorrhea, and hyperglycemia (13). Previous biological screenings revealed the numerous biological activities of *A. javanica*. Its ethanolic and aqueous extracts showed significant anti-diarrheal activity at a dose level of 800 mg/kg in Wistar rats (14). In addition, *A. javanica* exhibited anti-bacterial, anti-fungal (15–17), smooth muscle relaxant, and anti-ulcer activities (13). The alcoholic extract of root of *A. javanica* was shown to possess significant nephroprotective activity against cisplatin- and gentamycin-induced renal toxicity in experimental animals (18).

A phytochemical analysis of *A. javanica* has revealed the presence of polyphenols, terpenoids, flavonoids, and alkaloids (11). Notably, four new ecdysteroids, aervecdysteroid A–D (1–4), and three new acylated flavone glycosides have been isolated from the flowers (19). In addition, heptacosane (3-allyl-6-methoxyphenol) and pentacosane were identified as the major components of the *A. javanica* seed oil (20). With this background information, the present study was intended to investigate the antioxidant and hepatoprotective activities of ethanolic extract of the aerial parts of *A. javanica*.

## Materials and methods

### Plant materials

Aerial parts (leaves, stems, and inflorescences) of *A. javanica* (Family: *Amaranthaceae*) were collected from Taif, Kingdom of Saudi Arabia. The plant was authenticated by an expert plant taxonomist at the herbarium of College of Pharmacy, King Saud University, Riyadh, and a voucher specimen (No. 16281) was deposited.

### Preparation of *A. javanica* total ethanolic extract

The shade-dried powdered plant material (400 g) were soaked in 70% aqueous ethanol (Merck, Germany) for 2 days at room temperature and filtered. The extraction process was repeated twice with the same solvent. Then, the extracts were evaporated using a rotary evaporator (Buchi, Switzerland) under reduced pressure at 40°C. The obtained semi-solid extracts (37.68 g) were stored at −20°C until used for further study.

### Human hepatoma cell culture and drugs

Human hepatoma cell line (HepG2) was grown in RPMI-1640 medium, supplemented with 10% heat-inactivated bovine serum (Gibco, USA), 1× penicillin–streptomycin, and 1× sodium pyruvate streptomycin (HyClone Laboratories, USA) at 37°C in a humified chamber with 5% CO_2_ supply. 2,7-Dichlorofluorescein (DCFH; Sigma, USA) was used to induce cytotoxicity in cultured HepG2 cells. Silymarin (Sigma, USA) was used as standard hepatoprotective drug in rats.

### *Ex vivo* hepatoprotective assay of *A. javanica* total ethanolic extract

HepG2 cells were seeded (0.5×10^5^ cells/well, in triplicate) in a 96-well flat-bottom plate (Becton-Dickinson Labware, USA) and grown over night. The cytotoxicity of DCFH was determined by using MTT assay (MTT-Cell proliferation Assay Kit, Tervigen). The concentration of DCFH that caused a 50% inhibition of HepG2 cell proliferation (IC_50_: 100 µg/ml) was used as a cytotoxic dose. *A. javanica* total extract was dissolved in DMSO (100 mg/ml), followed by dilution with RPMI-1640 media to four doses (25, 50, 100, and 200 µg/ml). The final concentration of DMSO used never exceeded >0.1% and was therefore tolerated by cultured cells. The culture monolayer was replenished with RPMI-1640 containing 100 µg/ml DCFH plus a dose of *A. javanica*, including untreated as well as DCFH-treated controls. The treated cells were incubated for 48 h at 37°C. Cells were treated with MTT reagent (10 µl/well) and further incubated for 3–4 h. Upon the appearance of a purple color, detergent solution (100 µl) was added to each well and further incubated for 1.5 h. The optical density (OD) was recorded at 570 nm in a microplate reader (BioTek, ELx800). Nonlinear regression analysis was performed in Excel software to determine the cell survival (%) using the following equation:

$$\text{Celluar Survival}(\%)=\frac{\text{OD}[\text{s}]-\text{OD}[\text{b}]}{\text{OD}[\text{c}]-\text{OD}[\text{b}]}\times 100$$

where OD(s), OD(b), and OD(c) are the absorbance of sample, blank, and negative control, respectively.

### Microscopy

The morphological investigation of the cultured hepatoma cells was done under an inverted microscope (Optica, 40×, and 100×) to observe any changes in the cells cultured with different concentrations of *A. javanica* total extract and/or DCFH at 24 and 48 h.

### *In vivo* hepatoprotective activity of *A. javanica* total extract: Experimental design and treatment

Thirty healthy male Wistar rats were obtained from the Experimental Animal Care Center (EACC) of the College of Pharmacy, King Saud University, Riyadh. Animals were kept in polycarbonate cages in a room free from any source of contamination, artificially illuminated (12 h dark/light cycle) at controlled temperature (25±2°C). After acclimatization, animals were randomized and divided into five groups (I–V) of six animals each. Group I served as untreated control and fed orally with normal saline 1 ml. Group II received CCl_4_ in liquid paraffin (1:1) 1.25 ml/kgbw intraperitoneally (i.p.) and served as negative control. Similarly, group (III, IV and V) also received CCl_4_. While groups III and IV were treated with *A. javanica* total extract at a dose of 100 mg/kg.bw and 200 mg/kgbw, respectively, group V was treated with the standard drug silymarin (21–23) at a dose of 10 mg/kgbw for 3 weeks. Blood was collected for serum biochemistry and animals were sacrificed for liver dissection. All animals received human care in compliance with the guidelines of the Ethics Committee of the Experimental Animal Care Society, College of Pharmacy, King Saud University, Riyadh.

### Estimation of marker enzymes and bilirubin

Serum glutamate oxaloacetate transaminase (SGOT), serum glutamate pyruvate transaminase (SGPT) (24), alkaline phosphatase (ALP) (25), gamma-glutamyl transferase (GGT) (26), and bilirubin (27) were determined using Reflotron Plus Analyzer and Roche kits (Roche Diagnostics GmbH, Mannheim, Germany).

### Estimation of lipid profile

Total cholesterol (28), triglycerides (29) and high-density lipoproteins (HDL) (30) levels were estimated in serum using Roche diagnostic kits (Roche Diagnostics GmbH, Mannheim, Germany).

### Determination of malondialdehyde (MDA)

The method reported by Utley et al. (31) was followed. In brief, the liver tissues were removed homogenized in 0.15 M KCl (at 4°C; Potter-Elvehjem type C homogenizer) to give a 10% w/v homogenate. The absorbance of the solution was then read at 532 nm. The content of MDA (nmol/g wet tissue) was then calculated, by reference to a standard curve of MDA solution.

### Estimation of nonprotein sulfyhydryl (NP-SH)

Hepatic NP-SH was measured according to the method of Sedlak and Lindsay (32). The liver tissues were homogenized in ice-cold 0.02 mM ethylene diamine tetraacetic acid (EDTA). The absorbance was measured within 5 min of addition of 5,5′dithio-bis(2-nitrobenzoic acid) (DTNB) at 412 nm against a reagent blank.

### Determination of total protein (TP)

Serum TP was estimated by the kit method (Crescent Diagnostics, Jeddah, Saudi Arabia). The absorbance (Abs) of the samples were recorded at 546 nm and concentration of serum TP was calculated using the following equation:

(Serum TP=(Abs of sample/Abs of standard)×Concentration of standard)

### Histopathological evaluation

The animals were sacrificed under ethyl ether anesthesia and livers were quickly removed. Liver tissues were fixed in 10% neutral buffered formalin for 24 h and processed overnight for dehydration, clearing, and paraffin impregnation using an automatic tissue processor (Sakura, Japan). The specimens were embedded in paraffin blocks using embedding station (Sakura, Japan) and sections of 4 micron thickness were cut using rotary microtome (Leica-RM2245, Germany) and stained with hematoxylin and eosin (H&E) stains using routine procedures (33). The stained sections were observed under light microscopy, and the required images were taken with digital microscopic mounted camera (OMX1200C, Nikon, Japan).

### Antioxidant assay of *A. javanica* total extract

#### 1,1-Diphenyl-2-picrylhydrazyl (DPPH) radical scavenging assay

Antioxidant activity was estimated by free-radical scavenging ability of the total extract against DPPH according to the method described elsewhere (34), but with minor modifications to suite 96-well microplate format. DPPH is a molecule containing a stable free radical, and in the presence of an antioxidant agents, which can donate an electron to the DPPH, the purple color typical for free DPPH radical decays, and the change in absorbance (λ=517 nm) is measured spectrophotometrically. In brief, 100 µl of different concentrations (31.25, 62.5, 125, 250, and 500 µg/ml) of the extract was mixed with 40 µl of DPPH (0.2 mM in methanol) in wells of a 96-well microplate. Appropriate control was prepared using the solvent only in addition to the same amount of DPPH reagent to get rid of any inherent solvent effect. Ascorbic acid was used as standard. After 30 min incubation at 25°C, the decrease in absorbance was measured using microplate reader. The test was carried out in triplicate. The radical-scavenging activity was calculated from the equation:

$$\text{\% of radical scavenging activity}=[1-\frac{\text{Ab}{\text{s}}_{\text{sample}}}{\text{Ab}{\text{s}}_{\text{control}}}\times 100]$$

### β-Carotene-linoleic acid bleaching assay

The antioxidant activity of *A. javanica* total extract was evaluated using the β-carotene bleaching method (35) with minor modifications for working with 96 well plate. Briefly, 0.25 mg β-carotene was dissolved in 0.5 ml of chloroform and added to flasks containing 12.5 µg of linoleic acid and 100 mg of Tween-40. The chloroform was evaporated at 43°C using speed vacuum concentrator (Savant, Thermo Electron Co.). The resultant mixture was immediately diluted to 25 ml with distilled water and shaken vigorously for 2–3 min to form an emulsion. A 200 µl aliquot of the emulsion was added to wells of a 96-well plate containing 50 µl of the extract or gallic acid (500 µg/ml). A control containing solvent instead of extract was also prepared. The plate was incubated at 50°C for 2 h. Absorbance was read at 470 nm at 30 min intervals using microplate spectrophotometer (BioRad). The test was carried out in triplicate. The antioxidant activity was estimated using two different methods where the kinetic curve was initially obtained by plotting absorbance of each sample against time. Then, antioxidant activity was expressed as % inhibition of lipid peroxidation using the formula:

% Inhbibition=[(As(120)-Ac(120))/(Ac(0)-Ac(120))]×100

where As_(120)_ and Ac_(120)_ are the absorbance of the sample and control, respectively, at time 120 min, and AC(0) is the absorbance of the control at time 0 min.

### *In vitro* phytochemical screening of ethanolic extract of *A. javanica*

Qualitative phytochemical screening tests for major secondary metabolites, which included alkaloids, flavonoids, anthraquinones, tannins, saponins, and cardiac glycosides, were performed using standard procedures (36–38).

### Standardization of ethanolic extract of *A. javanica* by HPTLC method

A high-performance thin layer chromatography (HPTLC) method was used to standardize the 70% ethanol extract of *A. javanica* as described elsewhere (39). The chromatography was carried out on 10×10 cm precoated silica gel F_254_ RP-HPTLC plate, using rutin as the reference standard. Several mobile phases were tried to get good resolution and separation of different compounds present in the *A. javanica* ethanol extract. Based on our observations, we selected acetonitrile and water in the ratio of 4:6 as suitable mobile phase to carry out the standardization of the extract. The standard along with samples was applied on the HPTLC plate by CAMAG Automatic TLC Sampler-4. The plate was developed under controlled condition in CAMAG Automated Developing Chamber-2 and scanned by CAMAG TLC Scanner-3 (λ=360 nm).

### Statistical analysis

Results were expressed as mean±SEM. Total variation present in a set of data was estimated by one-way analysis of variance (ANOVA) followed by Dunnet's test. *P* <0.01 was considered significant.

## Results

### Effect of *A. javanica* on cell morphology

Microscopic observations showed considerable cytotoxic effect of DCFH on the HepG2 cells as reflected by altered morphology compared to untreated cells. Interestingly, the DCFH-treated cells supplemented with 100 and 200 µg/ml of *A. javanica* extract were morphologically different from those treated with DCFH alone but comparable to untreated cells (data not shown).

### Hepatoprotective and cell proliferative effect of *A. javanica*

**Fig. 1:**
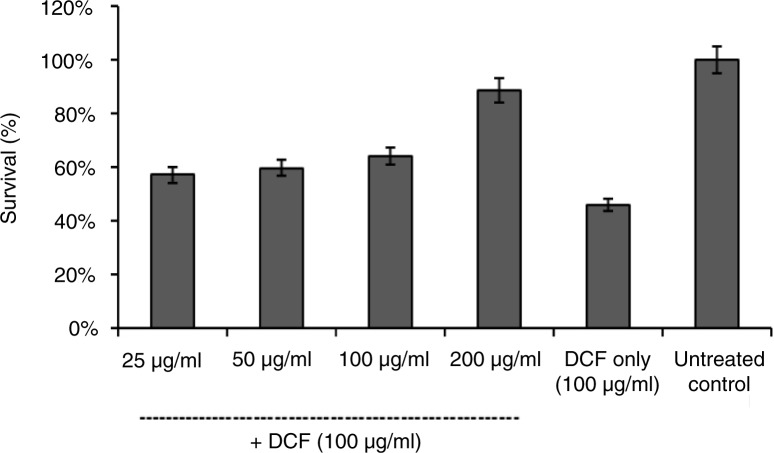
MTT-cell proliferation assay showing hepatoprotective effect of *A. javanica* total ethanolic extract against DCFH-induced hepatotoxicity in cultured HepG2 cells.

MTT test showed hepatoprotective effect of *A. javanica* ethanol extract in a dose-dependent manner against DCFH-toxicity. While DCFH-toxicated cells were recovered to about 56.7% after supplementation with 100 µg/ml of *A. javanica* extract, further doubling the dose (200 µg/ml) resulted in hepatocytes recovery by about 90.2% (Fig. 1).

### *In vivo* effect of *A. javanica* extract on biochemical markers

Based on the *ex vivo* hepatoprotective activity on HepG2 cells, the effects of *A. javanica* extract were further examined in the experimental animal model. The administration of CCl_4_ dramatically elevated the serum SGOT, SGPT, GGT ALP, and bilirubin levels compared to the normal control group (*P*<0.0001), indicating significant hepatotoxicity of CCl_4_ treatment (Table 1). In contrast, oral administration of *A. javanica* extract significantly normalized the biochemical parameters in CCl_4_-treated rats, compared to the CCl_4_-treated group (Table 1). Moreover, CCl_4_-induced toxicity caused significant elevation in lipid profile including cholesterol, triglycerides, low-density lipoprotein (LDL), and very-low-density lipoprotein (VLDL) and a reduction in the HDL levels in serum. The 3-week pretreatment of rats with two doses (250 and 500 mg/kg.bw/day) of *A. javanica* extract significantly reduced the cholesterol, triglycerides, LDL, and VLDL levels and improved the HDL level in a dose-dependent manner (Table 2). Silymarin, on the other hand, significantly prevented the CCl_4_-induced elevation in marker enzymes and lipid profile. Furthermore, our results indicated that treatment with *A. javanica* also resulted in a significant increase in MDA and decrease in NP-SH and TP concentration (Table 3). Oral supplementation of CCl_4_-treated rats with *A. javanica* extract dramatically diminished the levels of MDA and enhanced NP-SH and TP levels (Table 3).

**Table 1:** Effect of *A. javanica* total ethanolic extract on CCl_4_-induced hepatotoxicity-related parameters in rats

Treatment group	SGOT	SGPT (U/l)	ALP (U/l)	GGT (U/l)	Bilirubin (mg/dl)
Control	107.45±5.31	28.83±2.20	321.66±13.88	4.06±0.32	0.54±0.01
CCl_4_	294.83±8.33***^a^	230.83±9.62***^a^	515.16±13.70***^a^	12.85±0.98***^a^	2.16±0.08***^a^
*A. javanica* (100 mg) + CCl_4_	279.33±7.81^b^	174.33±6.13***^b^	437.00±8.66***^b^	10.28±0.43*^b^	1.81±0.05**^b^
*A. javanica* (200 mg) + CCl_4_	246.00±3.74***^b^	140.16±4.04***^b^	412.66±8.84***^b^	6.86±0.33***^b^	1.54±0.02***^b^
Silymarin (10 mg) + CCl_4_	136.66±6.00***^b^	85.66±4.31***^b^	396.33±7.62***^b^	5.58±0.28***^b^	1.06±0.06***^b^

All values represent mean±SEM

* *P*<0.05

** *P*<0.01

*** *P*<0.001; ANOVA, followed by Dunnet's multiple comparison test

a As compared with normal group.

b As compared with CCl_4_-only group.

**Table 2:** Effects of *A. javanica* total ethanolic extract on CCl_4_-induced lipid profile changes in rats

Treatment group	TC (mg/dl)	TG (mg/dl)	HDL (mg/dl)	LDL (mg/dl)	VLDL (mg/dl)
Control	109.83±3.94	59.01±2.74	55.18±2.40	42.84±3.22	11.80±0.54
CCl_4_	206.00±4.53***^a^	151.16±4.61***^a^	26.25±1.79***^a^	149.51±4.28***^a^	30.23±0.92***^a^
*A. javanica* (100 mg) + CCl_4_	163.33±5.28***^b^	105.85±5.20***^b^	29.76±1.02^b^	112.39±6.82***^b^	21.17±1.04***^b^
*A. javanica* (200 mg) + CCl_4_	143.66±5.47***^b^	82.61±3.65** b	35.41±2.26**^b^	91.72±6.25***^b^	16.52±0.70***^b^
Silymarin (10 mg) + CCl_4_	147.66±4.88***^b^	110.16±5.26***^b^	40.41±2.97**^b^	85.21±5.98***^b^	22.02±1.05***^b^

All values represent mean±SEM

* *P*<0.05

** *P*<0.01

*** *P*<0.001; ANOVA, followed by Dunnet's multiple comparison test.

a As compared with normal group.

b As compared with CCl_4_-only group.

**Table 3:** Biochemical parameters of rat liver tissues treated with *A. javanica* total ethanolic extract

Treatment group	MDA (nmol/g)	NP-SH (nmol/g)	TP (g/l)
Control	0.50±0.02	7.39±0.53	113.76±2.81
CCl_4_	4.82±0.29***^a^	3.86±0.44***^a^	49.11±1.82***^a^
*A. javanica* (100 mg) + CCl_4_	2.77±0.10***^b^	5.67±0.47*^b^	67.86±2.94***^b^
*A. javanica* (200 mg) + CCl_4_	1.79±0.14***^b^	6.16±0.40**^b^	82.23±4.44***^b^
Silymarin (10 mg) + CCl_4_	1.37±0.16***^b^	6.52±0.31***^b^	91.81±4.08***^b^

All values represent mean±SEM

* *P*<0.05

** *P*<0.01

*** *P*<0.001; ANOVA, followed by Dunnet's multiple comparison test.

a As compared with normal group.

b As compared with CCl_4_-only group.

### Histological improvement by *A. javanica* total extract

**Fig. 2:**
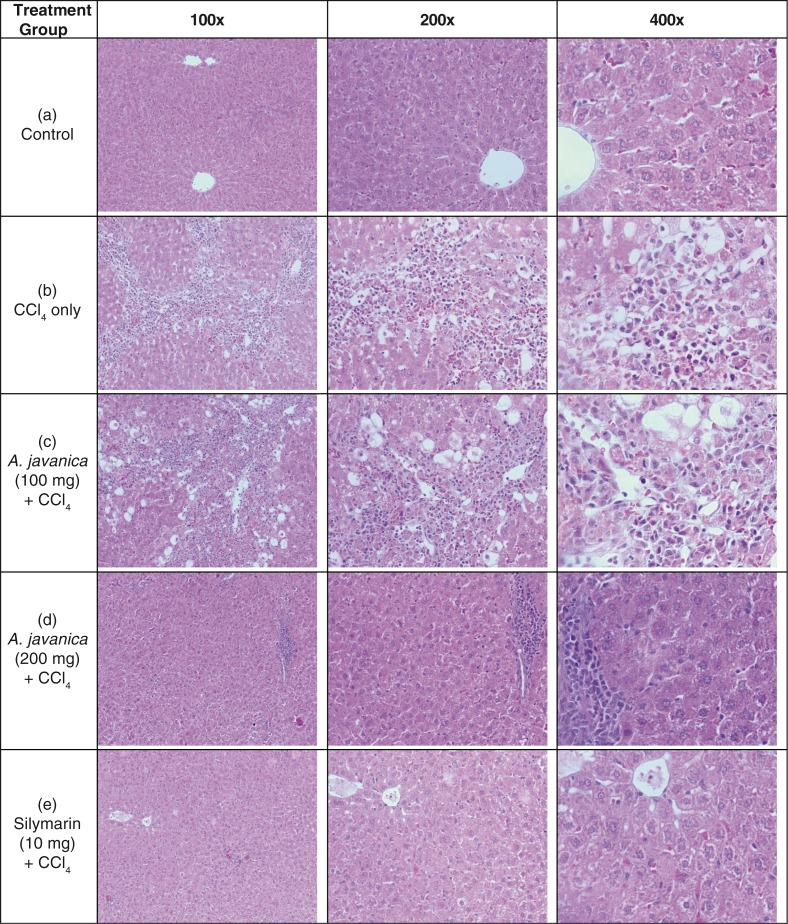
The histopathology of experimental rat liver at 100×, 200×, and 400× magnifications. Histograms showing the following: (a) healthy tissues with normal hepatocytes and central vein; (b) CCl_4_-injured tissue with necrosis and fatty degenerative changes; (c) tissue with congested central vein with necrosis and fatty changes after *A. javanica* (100 mg) + CCl_4_ treatment; (d) liver with normal hepatocytes and central vein with full recovery after *A. javanica* (200 mg) + CCl_4_ treatment; and (e) liver with normal hepatocytes and fully recovered central vein after silymarin (10 mg) + CCl_4_ treatment.

The histological examination of rat liver tissues revealed evidence of hepatic necrosis and fatty degenerative changes in CCl_4_-injured animals (Fig. 2a and b). Compared to this, the *A. javanica*-treated (100 mg/kg.bw/day) animals exhibited congested central vein with mild necrosis and fatty changes (Fig. 2c). On the other hand, the higher dose (200 mg/kg.bw/day) of *A. javanica* showed normal hepatocytes and central vein with full recovery that was comparable to silymarin administration (Fig. 2d and e). This finally confirmed the hepatoprotective efficacy of *A. javanica* total extract.

### In vitro antioxidant activity of *A. javanica*

**Fig. 3:**
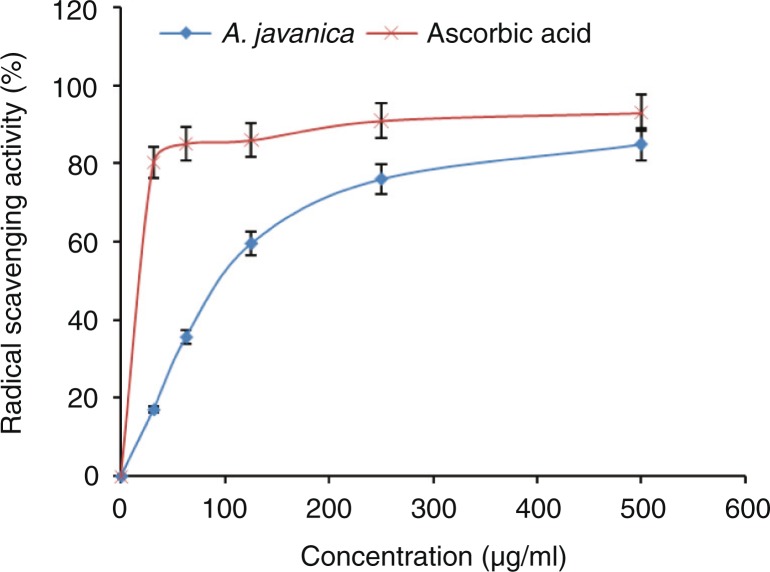
DPPH radical-scavenging activity of different concentrations (31.25–500 µg/ml) of total ethanolic extract of *A. javanica* and standard antioxidant (ascorbic acid).

**Fig. 4:**
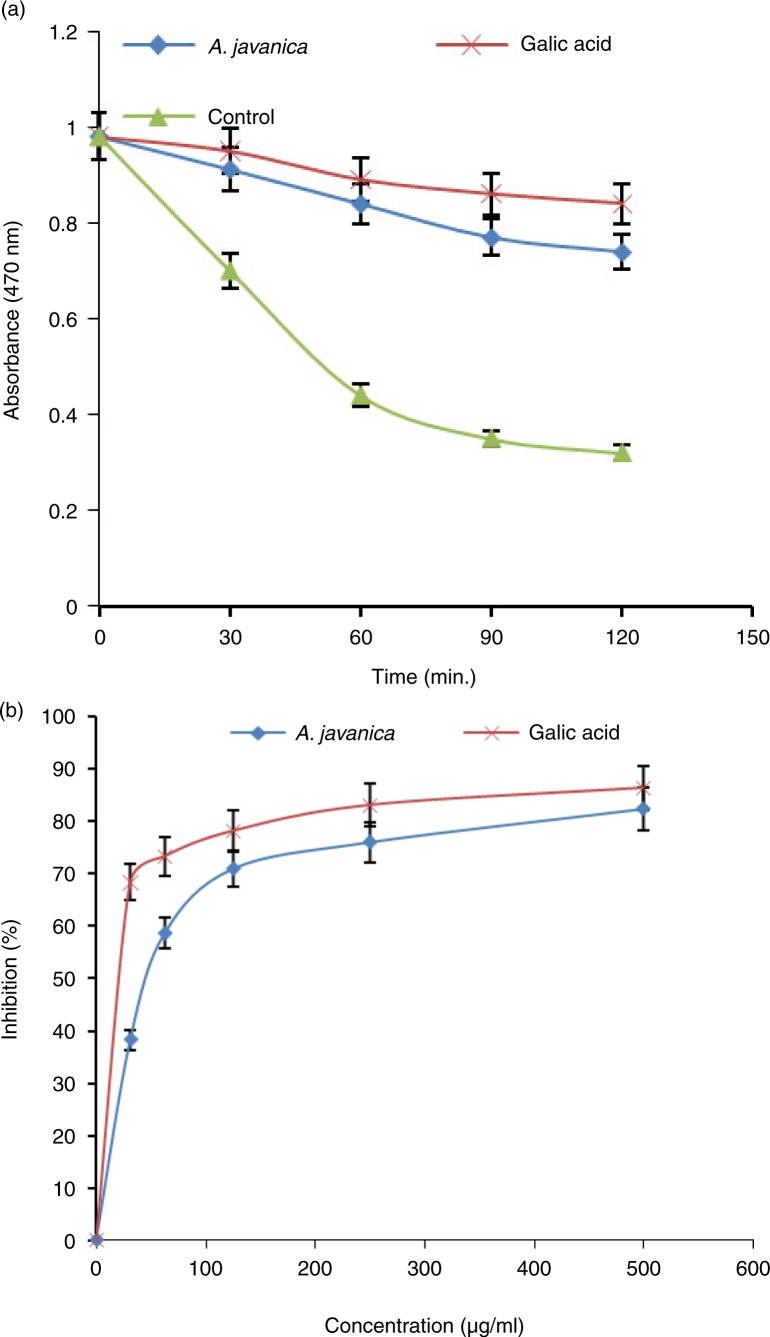
Antioxidative activity of *A. javanica* total ethanolic extract assayed by the β-carotene bleaching method. (a) β-carotene bleaching rate in the presence of 500 µg/ml of the extract, gallic acid (reference antioxidant), or blank control. (b) % inhibition of lipid peroxidation by different concentrations (31.25–500 µg/ml) of the extract and gallic acid.

The total extract of *A. javanica* was able to reduce the stable free-radical DPPH to the yellow-colored DPPH (Fig. 3). At 500 µg/ml dose, the observed antioxidant activities were comparable to that of the positive control (ascorbic acid). In accordance with the DPPH radical scavenging assay results, *A. javanica* showed high antioxidant activity in β-carotene–linoleic acid bleaching assay (Fig. 4).

### Phytochemical screening of the total extract

The qualitative phytochemical screening of the *A. javanica* total extract showed the presence of alkaloids, flavonoids, tannins, sterols, and saponins and the absence of anthraquinones and cardiac glycosides.

### Identification of rutin, a biflavonoid in *A. javanica* extract

**Fig. 5:**
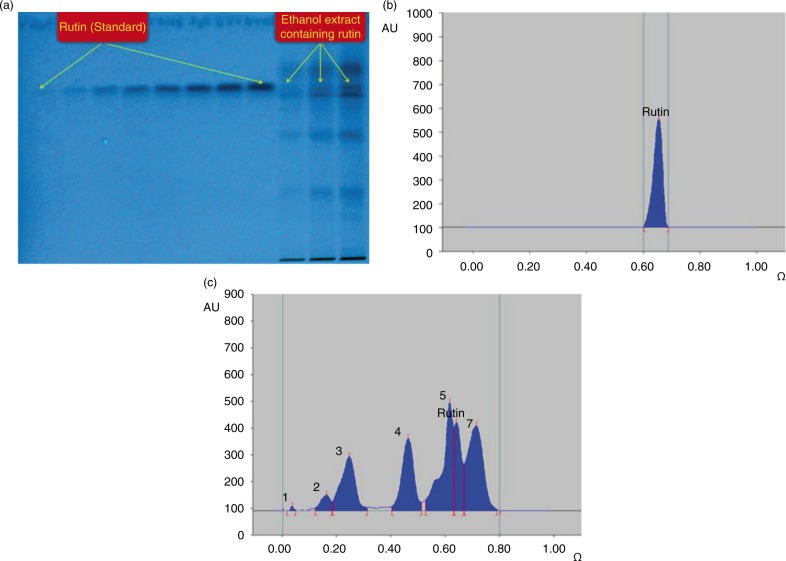
Quantification of rutin in *A. javanica* total ethanolic extract by HPTLC. (a) Pictogram of developed RP-HPTLC plate at 254 nm; mobile phase – acetonitrile: water (4:6, v/v). (b) Chromatogram of standard rutin (1,000 ng spot^−1^), peak 1 (*R*_f_=0.65) scanned at 360 nm; mobile phase – acetonitrile: water 4:6, v/v). (c) Chromatogram of the extract containing rutin (peak 6) scanned at 360 nm; mobile phase – acetonitrile: water 4:6, v/v).

The HPTLC analysis revealed the presence of rutin, a well-known antioxidant flavonoid in the total extract (Fig. 5a). Using acetonitrile and water as suitable mobile phase, the compact spot of rutin was found at *R*_f_=0.65 (Fig. 5b). Also, a good separation of different phytoconstituents present in the ethanol extract using the same mobile phase was achieved (Fig. 5c). The dried extract of *A. javanica* was found to contain 2.53 µg/mg of rutin.

## Discussion

Among more than 100 species of medicinal plants documented in Saudi Arabia, approximately 35 plants are used in Saudi folk medicine for the treatment of liver disorders. However, most of them remain popular without scientific rationale. Of these, the hepatoprotective potential of *Aerva javanica* has been neither explored in traditional practices nor with experimental approaches. In this study, we have, therefore, evaluated the hepatoprotective and antioxidative potential of *A. javanica* total ethanolic extract against DCFH-induced cytotoxicity in HepG2 cells and CCl_4_-induced liver damage in rats, respectively. DCFH is generally used for the quantitative assessment of oxidative stress generated by free radicals through the principle of oxidation of DCFH to the fluorescent DCF (40). However, we used this agent because of its toxic effect on cultured cell, *ex vivo*. *A. javanica* total extract promoted HepG2 cell proliferation and recovery in a dose-dependent manner. These findings were further supported by microscopic examination of cell morphology and growth.

To further confirm the *ex vivo* cell proliferative effects, the *in vivo* hepatoprotective potential activity of *A. javanica* total extract was examined in CCl_4_-injured rats. CCl_4_ is a common hepatotoxin used in the experimental study of liver diseases that induces free-radical generation in liver tissues. *In vivo*, it is transformed to active free radicals that bind to macromolecules resulting in lipid peroxidation that causes cell injury (41, 42). Clinical symptoms of CCl_4_-induced acute liver injury include jaundice and elevated levels of serum SGOT, SGPT, and ALP, indicators of liver necrosis and inflammation (43). In the present study, pretreatment with two doses of *A. javanica* total extract (100 and 200 mg/kg.bw) showed its ability to reduce the SGOT, SGPT, GGT, ALP, and bilirubin levels significantly in a dose-dependent manner. In addition, *A. javanica* extract was able to normalize the serum cholesterol and triglycerides and HDL levels in CCl_4_-injured rats. The hepatoprotective effect of *A. javanica* at 200 mg/kg.bw was comparable to standard drugs silymarin (10 mg/kg.bw). The rise of serum enzyme SGOT and SGPT levels had indicated that hepatocyte cells were injured where the leakage of cell membrane had contributed to the accumulation of these enzymes into the plasma (44). The significant reduction in the levels of LDL, and total cholesterol in the *A. javanica-*treated rats and an increase in the HDL level further indicated the hepatoprotective potential of *A. javanica*.

MDA is produced during the peroxidation of polyunsaturated fatty acids, and the amount of MDA is a marker for cell membrane lipid peroxidation and cell damage (45). Treatment with *A. javanica* extract or silymarin reduced the level of MDA in livers with CCl_4_-induced damage, which suggests that the tested extract was protective and curative against liver toxicity.

NP-SH plays an important role in the defense against oxidative cellular damage (46). In our study, the liver NP-SH level in the CCl_4_-treated group was markedly reduced when compared with the control group. Pretreatment of rats with *A. javanica* total extract or silymarin replenished NP-SH concentration as compared with CCl_4_ only treated animals, suggesting free-radical scavenging activity of the tested extract.

The level of TP would be decreased in hepatotoxic conditions due to defective protein biosynthesis in liver. Thus, the reduction in TP level is a further indication of liver damage in CCl_4_-injured animals (47). In our study, the level of TP has been restored to the normal value, indicating the curative action of *A. javanica* total ethanolic extract. Moreover, most of the parameters in the animal group that received CCl_4_ plus *A. javanica* extract had values comparable to those of the group receiving CCl_4_ plus silymarin. This very clearly indicated that *A. javanica* was efficient to attenuate CCl_4_-induced hepatotoxicity in rats.

Conducting different assay methods for the evaluation of antioxidant activity (48, 49) is now commonly recommended. In our *in vitro* assays, the antioxidant activity of *A. javanica* ethanolic extract revealed strong antioxidant activities in both DPPH and β-carotene methods. In addition, our preliminary phytochemical screening of the *A. javanica* total extract showed the presence of alkaloids, flavonoids, tannins, sterols, and saponins. Taken together, the antioxidant activity of the *A. javanica* extract could be attributed to the presence of antioxidant and free-radical scavenging phytoconstituents, such as polyphenol, flavonoids, and saponins, which are known to have hepatoprotective activities. Rutin (3,3′,4′,5,7-pentahydroxyflavone-3-rhamnoglucoside), a biflavonoid found in many plants, has a wide range of pharmacological properties against various metabolic and infectious diseases (50). The reactive-species-mediated *in vivo* hepatotoxicity can be effectively attenuated by rutin possessing antioxidant, free-radical scavenger, and anti-lipid per oxidant activities (51). Recently, hepatoprotective mechanisms of rutin in CCl_4_-intoxicated BALB/cN mice has also been demonstrated (52). Our identification of rutin in *A. javanica* extract by validated HPTLC method further supports its therapeutic use in the prevention and treatment of liver diseases.

Very recently, *A. Javanica*-derived aervecdysteroid A–D (1–4) and acylated flavone glycosides have been shown to inhibit enzymatic activities of lipoxygenase and butyrylcholinesterase (53). In mammals, lipoxygenases catalyze the formation of hydroperoxides as the first step in the biosynthesis of several inflammatory mediators (54). Butyrylcholinesterase, also known as serum or plasma cholinesterase, is synthesized in the liver that hydrolyzes many different choline-based esters. In clinical assays, serum butyrylcholinesterase activity is used as a liver function test for acute and chronic liver damage, including other inflammatory disorder (55). Nevertheless, further biological analysis of these compounds, including identification of other bioactive constituents of *A. javanica* with antioxidative and hepatoprotective activities, remains to be explored.

## Conclusions

Our investigation of *A. javanica* total ethanol extract revealed its promising antioxidative and hepatoprotective potential against chemically induced liver injury *ex vivo* as well as *in vivo*. This was supported by the *in vitro* phytochemical analysis and identification of rutin, a well-known antioxidant flavonoid. Our finding therefore, suggests the therapeutic potentiality of *A. javanica* in various liver diseases. However, further studies on main active ingredients of the extract, their mechanism of action, and other therapeutic contribution of these interventions would be necessary.
